# The 5-HT_7_ receptor antagonist SB 269970 ameliorates corticosterone-induced alterations in 5-HT_7_ receptor-mediated modulation of GABAergic transmission in the rat dorsal raphe nucleus

**DOI:** 10.1007/s00213-018-5045-y

**Published:** 2018-09-28

**Authors:** Joanna Sowa, Magdalena Kusek, Marcin Siwiec, Joanna Ewa Sowa, Bartosz Bobula, Krzysztof Tokarski, Grzegorz Hess

**Affiliations:** Department of Physiology, Institute of Pharmacology, Polish Academy of Sciences, Smetna 12, 31-343 Krakow, Poland

**Keywords:** 5-HT_7_ receptor, SB 269970, Stress, Corticosterone, Dorsal raphe nuclei, GABAergic transmission, Serotonin

## Abstract

**Rationale:**

Chronic stress and corticosterone have been shown to affect serotonin (5-HT) neurotransmission; however, the influence of stress on the activity of the dorsal raphe nucleus (DRN), the main source of 5-HT in the forebrain, is not well understood. In particular, it is unknown if and how stress modifies DRN 5-HT_7_ receptors, which are involved in the modulation of the firing of local inhibitory interneurons responsible for regulating the activity of DRN projection cells.

**Objectives:**

Our study aimed to investigate the effect of repeated corticosterone injections on the modulation of the inhibitory transmission within the DRN by 5-HT_7_ receptors and whether it could be reversed by treatment with a 5-HT_7_ receptor antagonist.

**Methods:**

Male Wistar rats received corticosterone injections repeated twice daily for 14 days. Spontaneous inhibitory postsynaptic currents (sIPSCs) were then recorded from DRN projection cells in ex vivo slice preparations obtained 24 h after the last injection.

**Results:**

Repeated corticosterone administration resulted in decreased frequency, but not amplitude, of sIPSCs in DRN projection cells. There were no changes in the excitability of these cells; however, corticosterone treatment suppressed the 5-HT_7_ receptor-mediated increase in sIPSC frequency. Administration of the 5-HT_7_ receptor antagonist SB 269970 for 7 days beginning on the eighth day of corticosterone treatment reversed the detrimental effects of corticosterone on 5-HT_7_ receptor reactivity and GABAergic transmission in the DRN.

**Conclusions:**

Elevated corticosterone level reduces DRN 5HT_7_ receptor reactivity and decreases GABAergic transmission within the DRN, which can be reversed by the 5-HT_7_ receptor antagonist SB 269970.

## Introduction

Chronic stress and elevated blood cortisol levels are risk factors causally related to the development of a number of human pathologies including depressive disorders (reviewed in: Claes 2004; Parker et al. 2003; Reagan et al. 2008). It has been established that the pathomechanism of depressive disorders involves dysfunctions of the brain serotonin (5-hydroxytryptamine, 5-HT) system (reviewed in: Stahl 1998; Köhler et al. 2016). Animal studies have shown that both acute and chronic stressors affect 5-HT neurotransmission, evoking alterations in 5-HT release and reuptake as well as changes in extracellular 5-HT and 5-HT receptors’ levels (Adell et al. 1988; reviewed in: Chaouloff 2000).

The dorsal raphe nucleus (DRN) is the main source of widespread 5-HT innervation, which modulates the activity of neuronal networks in target forebrain structures via several 5-HT receptor subtypes (reviewed in: Jacobs and Azmitia 1992; Celada et al. 2013). 5-HT may also influence the activity of DRN 5-HT neurons themselves as these cells express 5-HT_1A_, 5-HT_1B_, 5-HT_1D_, and possibly 5-HT_2_ autoreceptors (McDevitt and Neumaier 2011). 5-HT neurons of the DRN are known to express glucocorticoid receptors (GRs; Harfstrand et al. 1986). Chronic exposure to elevated corticosterone levels and uncontrollable stress have been demonstrated to induce a functional desensitization of 5-HT_1A_ somatodendritic autoreceptors (Fairchild et al. 2003; Rainer et al. 2012; Rozeske et al. 2011; reviewed in: McDevitt and Neumaier 2011), which normally effectively attenuate spiking activity of DRN 5-HT projection neurons (Aghajanian et al. 1990). Apart from 5-HT projections cells, the DRN contains a population of GABAergic interneurons which provide inhibitory input onto DRN projection cells. These inhibitory interneurons express 5-HT_2A/C_ and possibly 5-HT_1A_ and 5-HT_6_ receptors (Asaoka et al. 2015; Gocho et al. 2013; Liu et al. 2000). Another serotonin receptor abundant in the DRN is the 5-HT_7_ receptor (Roberts et al. 2004). Its role in the regulation of the activity of DRN neuronal network in normal and pathological conditions is not completely understood. Importantly, 5-HT_7_ receptors in the DRN are present on GABAergic interneurons, but not on 5-HT cells (Monti et al. 2008; Kusek et al. 2015). 5-HT_7_ receptor activation increases the mean frequency of spontaneous inhibitory postsynaptic currents (sIPSCs) recorded from DRN projection cells, as well as induces their hyperpolarization and decreases their firing frequency (Kusek et al. 2015). Systemic blockade of 5-HT_7_ receptors enhances 5-HT transmission and metabolism in target structures (Mnie-Filali et al. 2011; Kusek et al. 2015). While modifications of the GABAergic input onto DRN 5-HT neurons have been shown to play an important role in facilitating certain stress-induced behaviors (Challis et al. 2013; Crawford et al. 2013), the influence of prolonged elevation of corticosterone level on GABAergic transmission within the DRN and its modulation by the 5-HT_7_ receptor has not yet been investigated. Repeated corticosterone administration has been proposed as a preclinical rat model to study links between stress, glucocorticoids, and depressive behavior (reviewed in: Sterner and Kalynchuk 2010). In the present study, we aimed at determining the effects of corticosterone treatment lasting 14 days on the ability of the 5-HT_7_ receptor to modulate inhibitory transmission in the DRN.

5-HT_7_ receptor blockade has been shown to induce antidepressant-like effects in animal models (reviewed in: Ciranna and Catania 2014; Nikiforuk 2015). These effects occur faster than those induced by conventional antidepressant drugs (Mnie-Filali et al. 2011; reviewed in: Tokarski et al. 2012a). We have previously demonstrated that treatment with the selective 5-HT_7_ receptor antagonist SB 269970 counteracted repeated restraint stress-induced modifications of glutamatergic transmission and synaptic plasticity in the rat frontal cortex (Tokarski et al. 2011). However, the plausible influence of 5-HT_7_ receptor antagonism on the effects of prolonged corticosterone treatment on functional properties of DRN neurons has not yet been investigated. Therefore, the second aim of the present study was to determine whether blocking 5-HT_7_ receptors with SB 269970 could reverse corticosterone-induced alterations in 5-HT_7_ receptor-dependent modulation of GABAergic transmission in the DRN.

## Experimental procedures

### Animals

The experimental procedures were approved by the Local Ethics Committee for Animal Experiments at the Institute of Pharmacology, Polish Academy of Sciences, and were carried out in accordance with the European Community guidelines for the use of experimental animals and the national law. Male Wistar rats (Charles River, Germany) weighing approx. 150 g at the beginning of the experiment were housed in standard laboratory cages and maintained on a 12-h light/dark schedule (lights on at 07:00 and off at 19:00) with free access to standard food and tap water.

### Treatment

The animals were assigned to four groups. In the first experimental group (termed: Cort + SB), animals received corticosterone injections subcutaneously (dose 10 mg/kg, volume 1 ml/kg; in 1% solution of Tween 80 in water; Zahorodna and Hess 2006) twice daily for 14 days and, beginning on the eighth day of corticosterone treatment, additionally the 5-HT_7_ receptor antagonist, SB 269970 (Tocris) for 7 days. SB 269970 was dissolved in 0.9% NaCl and injected intraperitoneally (dose 2.5 mg/kg, volume 1 ml/kg) once daily. The second group (termed: Cort + NaCl) received corticosterone for 14 days and, beginning on the eighth day of corticosterone treatment, injections of 0.9% NaCl for 7 days. Control rats for these treatments received 1% Tween 80 for 14 days and SB 269970 for 7 days (termed: Tween + SB) or 1% Tween 80 for 14 days and 0.9% NaCl for 7 days (termed: Tween + NaCl). The number of animals in each group was 10.

### Slice preparation and incubation

Brain slices were prepared 24 h after the last substance administration to avoid acute effects of corticosterone and SB 269970 (Hagan et al. 2000; Droste et al. 2008). Rats were anesthetized with isoflurane (Aerrane, Baxter, UK) and decapitated. Their brains were quickly removed and immersed in an ice-cold artificial cerebrospinal fluid (ACSF) containing (in mM) NaCl (130), KCl (5), CaCl_2_ (2.5), MgSO_4_ (1.3), KH_2_PO_4_ (1.25), NaHCO_3_ (26), and d-glucose (10), bubbled with a mixture of 95% O_2_–5% CO_2_. Coronal midbrain slices containing the DRN (thickness 300 μm) were cut using a vibrating microtome (Leica VT1000) and subsequently stored submerged in ACSF at 30 ± 0.5 °C. Two or three slices were obtained from each animal.

### Whole-cell recording

After at least 3 h of preincubation, an individual slice was placed in the recording chamber and superfused without recycling at 2.5 ml/min with warm (32 ± 0.5 °C; Bacon and Beck 2000, Liu et al. 2000), modified ACSF of the following composition (in mM): NaCl (132), KCl (2), CaCl_2_ (2.5), MgSO_4_ (1.3), KH_2_PO_4_ (1.25), NaHCO_3_ (26), and d-glucose (10), bubbled with 95% O_2_–5% CO_2_. Modified ACSF also contained *N*-[2-[4-(2-methoxyphenyl)-1-piperazinyl]ethyl]-*N*-2-pyridinylcyclohexanecarboxamide (WAY 100635, Tocris, 2 μM**)** to block 5-HT_1A_ receptors, as during the recordings, 5-carboxyamidotryptamine (5-CT, Tocris; 250 nM), a nonselective 5-HT_1A_ and 5-HT_7_ receptor agonist, was used to activate 5-HT_7_ receptors.

Whole-cell recordings were obtained from the dorsal part of the midline region of the DRN. Neurons were visualized using the Zeiss Axioscope 2 upright microscope (Nomarski optics), a × 40 water immersion objective and an infrared camera. Recording pipettes were pulled from borosilicate glass capillaries (Harvard Apparatus), using the Sutter Instrument P97 puller. The pipette solution contained (in mM) K-gluconate (130), NaCl (5), CaCl_2_ (0.3), MgCl_2_ (2), HEPES (10), Na_2_-ATP (5), Na-GTP (0.4), and EGTA (1). Osmolarity and pH were adjusted to 290 mOsm and 7.2, respectively. Pipettes had an open tip resistance of approx. 6 MΩ. Signals were recorded using the SEC 05LX amplifier (NPI, Germany), filtered at 2 kHz and digitized at 20 kHz using a Digidata 1440A interface and Clampex 10 software (Molecular Devices, USA).

Putative 5-HT DRN projection neurons were identified by their response to hyper- and depolarizing current pulses (duration 400 ms; Galindo-Charles et al. 2008; Kusek et al. 2015). For each cell, the relationship between the injected current intensity and the number of spikes was plotted and gain was determined as the slope of a straight line fitted to experimental data.

Cells were voltage-clamped at 0 mV, and after 15 min of stabilization sIPSCs were recorded for 4 min as outwards currents. We have previously shown that these currents could be blocked by bicuculline (Kusek et al. 2015). Next, 5-carboxyamidotryptamine (5-CT, Tocris; 250 nM) was added to the ACSF (Kusek et al. 2015). Following 15 min of stabilization, sIPSCs were again recorded for 4 min. Data were accepted for analysis when the access resistance ranged between 15 and 18 MΩ and remained stable (< 25% change) during the recording.

Spontaneous IPSCs were detected offline using Mini Analysis software (Synaptosoft), and individual synaptic events were manually selected for further analysis. The threshold amplitude for the detection of an IPSC was set to 10 pA. IPSC kinetics were determined from the averaged IPSC for each cell. Rise time was measured as the time needed for the current to rise from 10 to 90% of the peak. The decay time constant (tau) was determined from fitting an exponential function to the decay phase of the IPSC.

### Statistical analysis

Rats were weighed on a daily basis. Average growth curves for each experimental treatment group were constructed by fitting linear regression lines to raw data. Slopes for the individual fits were compared across groups using the two-way analysis of variance (ANOVA), followed by Sidak’s multiple comparisons test. Statistical analyses of the electrophysiological data were carried out using two-way ANOVA followed by Sidak’s multiple comparison test. For estimating the effects of 5-CT on synaptic activity in brain slices from the different treatment groups, the percentage change relative to baseline was used as the dependent variable. The analysis was performed in GraphPad Prism 7 software. The results are expressed as the mean ± SEM. The significance level was set at *p* = 0.05 for all comparisons.

## Results

### SB 269970 does not change the effect of corticosterone on animal body weight

**Fig. 1:**
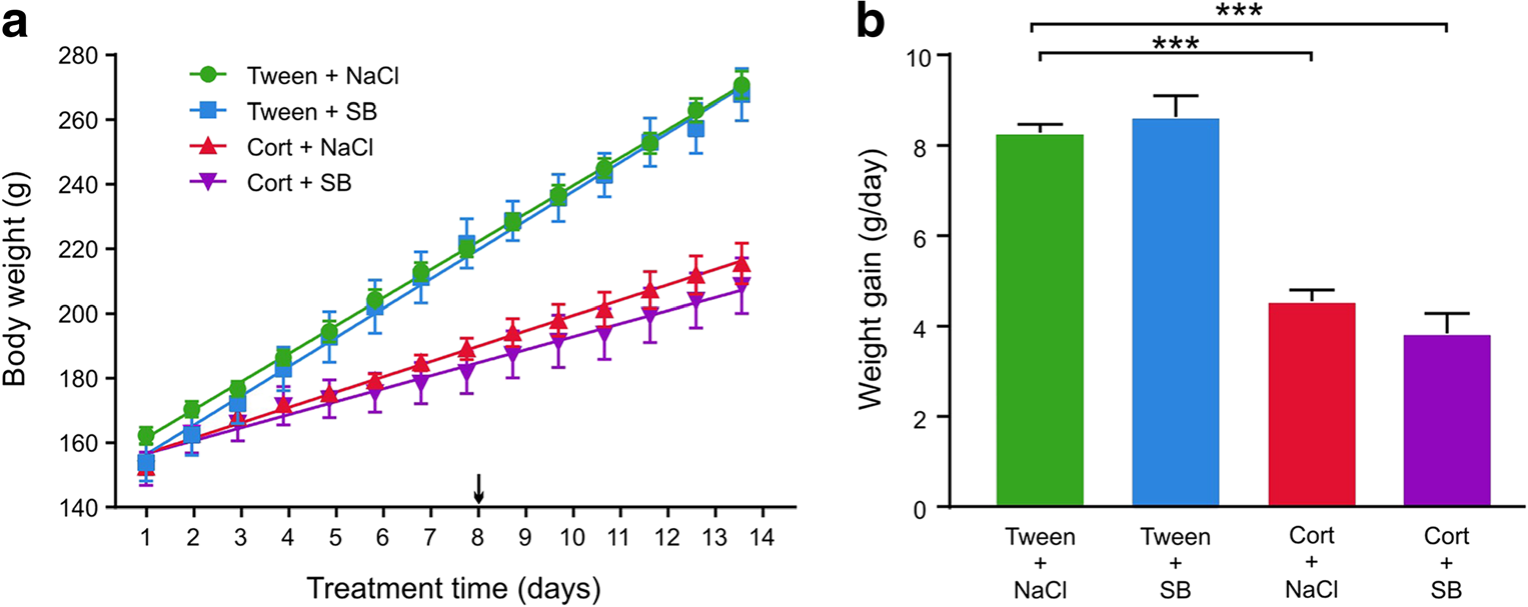
Effects of repeated corticosterone and 5-HT_7_ receptor antagonist SB 269970 injections on animal body weight. Rats from the Cort + NaCl group, as well as from the Cort + SB group, gained significantly less weight compared to control (Tween + NaCl) animals. Concurrent administration of SB 269970 and corticosterone (Cort + SB) did not modify the effect of corticosterone on body weight gain (measured as the slope of the linear regression line). No differences were evident between the Tween + SB and the Tween + NaCl group. The number of animals in each group was 10. Data are mean ± SEM. ***p < 0.001. The arrow indicates the first day of SB 269970 injections

Rats from the Cort + NaCl group gained significantly less weight compared to the animals receiving the vehicle (Tween + NaCl group; Fig. 1). Concurrent administration of SB 269970 and corticosterone (Cort + SB group) did not modify the effect of corticosterone on body weight gain (measured as the slope of the linear regression line). No differences were evident between rats receiving the vehicle and SB 269970 injections (Tween + SB group) and the Tween + NaCl group. A significant main effect of corticosterone on body weight gain (*F*_(1, 36)_ = 147.1, *p* < 0.0001) was observed. Animals from the Cort + NaCl and Cort + SB groups gained weight significantly slower than Tween + NaCl receiving rats (*p* < 0.0001, Sidak’s multiple comparisons test).

### Lack of effect of corticosterone and SB 269970, alone and in combination, on DRN neuronal excitability

**Fig. 2:**
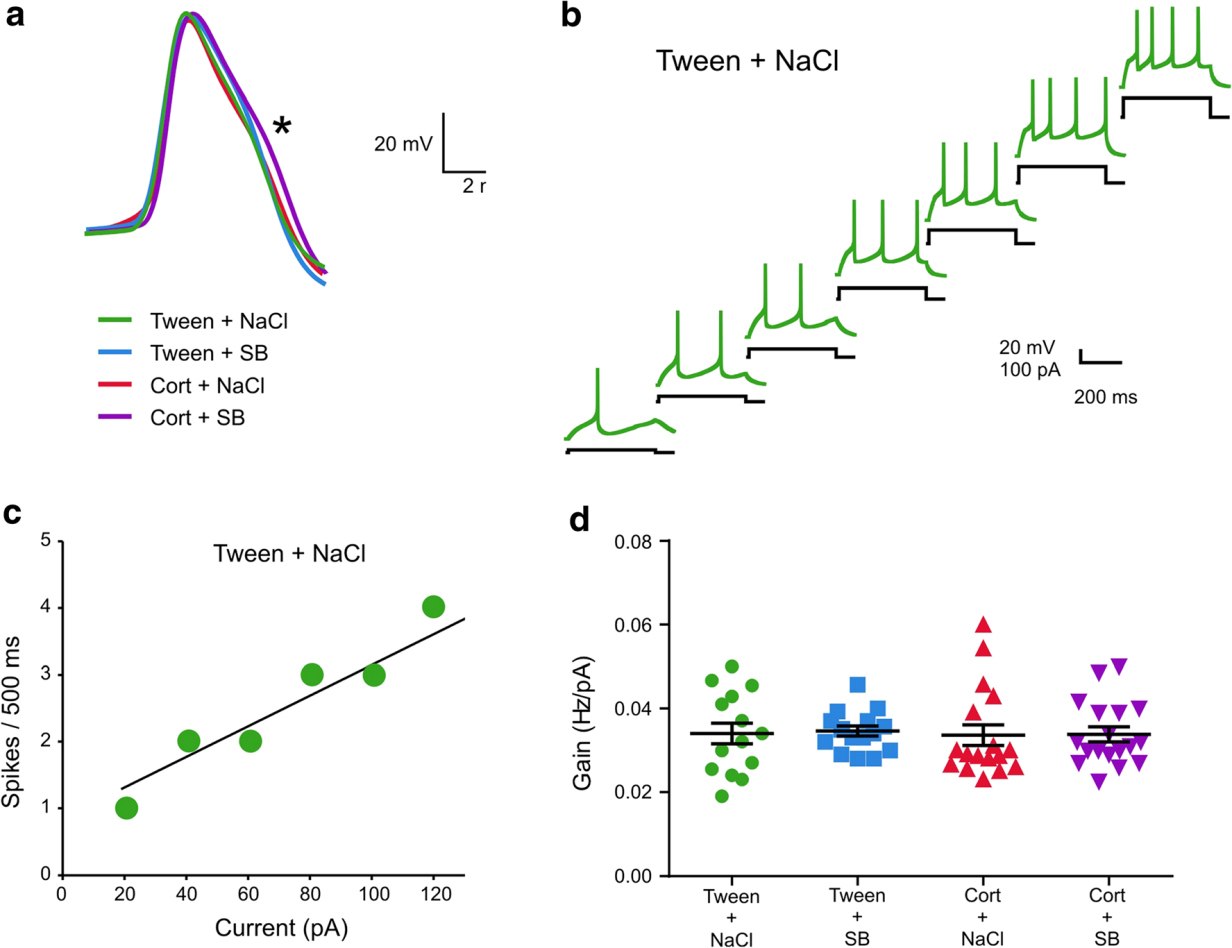
Repeated corticosterone and SB 269970 injections do not influence basic electrophysiological properties and excitability of DRN projection neurons. **a** Examples of single action potentials from all examined groups, with the “notch” on their descending phase marked with an asterisk. **b** Responses of representative projection neuron to different current injections (step: 20 pA) recorded in DRN slice prepared from control (Tween + NaCl) animal. **c** Relationship between spiking rate and injected current for the cell shown in panel **b**. The slope of the straight line fitted to experimental data represents gain. **d** Summary graph showing the mean gain (± SEM) of all neurons from the Tween + NaCl, Tween + SB, Cort + NaCl, and Cort + SB-treated rats. The differences between groups are not significant

All cells subjected to analysis, when stimulated by depolarizing current pulses fired broad action potentials with a “notch” on their descending phase (Fig. 2), characteristic of putative 5-HT projection neurons (see also: Galindo-Charles et al. 2008; Kusek et al. 2015). The resting membrane potential and input resistance of recorded DRN neurons did not differ significantly between groups (Table 1). In addition, there were no differences in neuronal excitability between any groups.

**Table 1:** Effects of the treatments on basic electrophysiological characteristics of recorded neurons (mean ± SEM)

Group	*V*_m_ (mV)	*R*_m_ (MΩ)	Gain (Hz/pA)	*n*
Tween + NaCl	− 67.86 ± 1.34	593.70 ± 29.12	0.035 ± 0.003	14
Tween + SB	− 67.60 ± 0.88	578.70 ± 18.24	0.035 ± 0.001	15
Cort + NaCl	− 69.00 ± 1.14	591.70 ± 17.96	0.034 ± 0.003	17
Cort + SB	− 64.78 ± 1.30	592.80 ± 18.61	0.034 ± 0.002	18

Differences between values are not significant (*p* > 0.05)

*V*_m_ resting membrane potential, *R*_m_ input resistance, *n* number of cells

### SB 269970 reverses effects of corticosterone treatment on the inhibitory input to DRN neurons

**Fig. 3:**
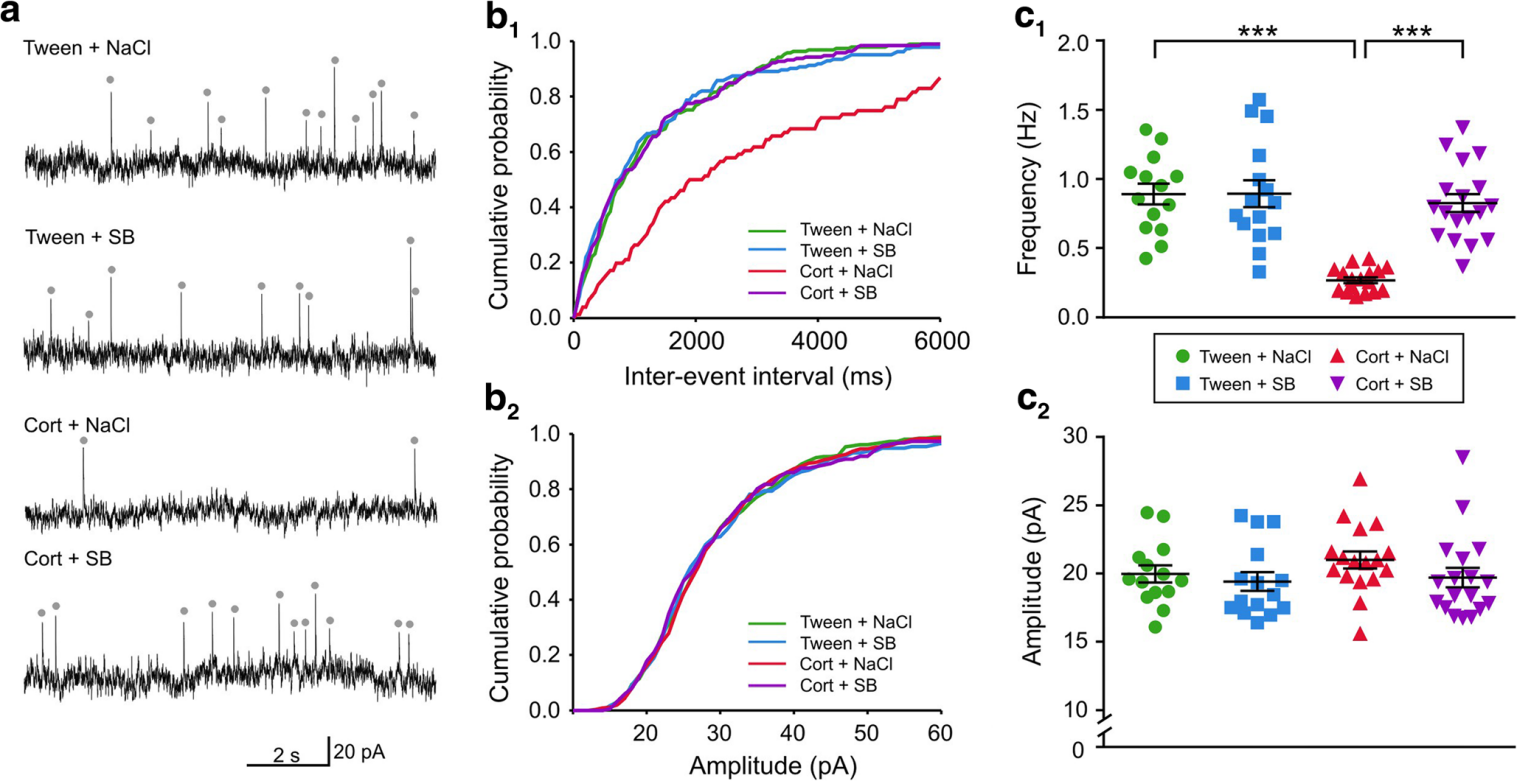
SB 269970 reverses the effect of repeated corticosterone administration on DRN GABAergic transmission. **a** Sample recordings from representative neurons in slices prepared from animals treated with Tween + NaCl (*first trace*), Tween + SB (*second trace*), Cort + NaCl (*third trace*), and Cort + SB (*fourth trace*). Dots mark spontaneous synaptic events. (**b**_**1**_) Cumulative probability plots of inter-event intervals of sIPSCs recorded from individual representative neurons from all four groups of rats. **b**_**2**_ Cumulative probability plots of amplitudes of sIPSCs recorded from individual representative neurons. **c**_**1**_ Summary graph showing the mean frequency (± SEM) of sIPSCs recorded from all neurons from the Tween + NaCl, Tween + SB, Cort + NaCl, and Cort + SB-treated rats. ***p < 0.001. **c**_**2**_ A comparison of the mean amplitude (± SEM) of sIPSCs recorded from all neurons of the four investigated groups of animals. Labels as in panel **c**_**1**_

There was a significant effect of corticosterone treatment (*F*_(1, 60)_ = 14.66, *p* = 0.0003), SB 269970 treatment (*F*_(1, 60)_ = 15.31, *p* = 0.0002), and their interaction (*F*_(1, 60)_ = 14.99, *p* = 0.0003) on the frequency of sIPSCs. The sIPSC frequency was lower in the group receiving corticosterone and 0.9% NaCl injections (Cort + NaCl) compared to the Tween + NaCl group (0.27 ± 0.02 Hz vs. 0.82 ± 0.08 Hz, respectively; *n* = 17 and 14, *t* = 5.361, *df* = 60, *p* < 0.0001; Sidak’s multiple comparison test), as well as when compared to the Cort + SB group (0.27 ± 0.02 Hz vs. 0.83 ± 0.06 Hz, respectively; *n* = 17 and 18, *t* = 5.783, *df* = 60, *p* < 0.0001; Sidak’s multiple comparison test; Fig. 3b_1_, c_1_). No differences between the Cort + SB and Tween + NaCl groups were observed (0.83 ± 0.06 vs. 0.82 ± 0.08, respectively; *n* = 18 and 14, *t* = 0.059, *df* = 60, *p* = 0.9999; Sidak’s multiple comparison test). There were no significant effects of SB 269970 injections (Tween + SB) on sIPSC frequency compared to control (Tween + NaCl) (0.82 ± 0.10 vs. 0.82 ± 0.08, respectively; *n* = 15 and 14, *t* = 0.028, *df* = 60, *p* = 0.9999; Sidak’s multiple comparison test).

The analysis did not reveal any effect of treatments (corticosterone treatment: *F*(_1, 60)_ = 0.8172, *p* = 0.3696; SB 269970 treatment: *F*_(1, 60)_ = 1.851, *p* = 0.1787) or their interaction (*F*_(1, 60)_ = 0.2929, *p* = 0.5903) on sIPSC amplitude (Fig. 3b_2_, c_2_).

No significant effect of SB 269970 treatment (*F*_(1, 60)_ = 0.4056, *p* = 0.5266) and interaction (*F*_(1, 60)_ = 0.1992, *p* = 0.6569), but significant effect of corticosterone treatment (*F*_(1, 60)_ = 6.739, *p* = 0.0118) on the rise time of sIPSCs was observed. However, multiple comparisons with Sidak’s test did not reveal any significant differences between groups. A significant effect of treatment with SB 269970 (*F*_(1, 60)_ = 5.292, *p* = 0.0249) on the decay time constant of sIPSCs and interaction (*F*_(1, 60)_ = 3.877, *p* = 0.0536) with no effect of corticosterone treatment (*F*_(1, 60)_ = 1.929, *p* = 0.1700) was observed (Table 2). The decay time constant was found to be shorter in the group of animals receiving Tween and SB 269970 injections compared to control (6.58 ± 0.19 vs. 7.46 ± 0.17, respectively; *n* = 15 and 14, *t* = 2.886, *df* = 60, *p* = 0.032; Sidak’s multiple comparison test).

**Table 2:** Effects of corticosterone and SB 269970 treatment on sIPSC characteristics (mean ± SEM) in the four tested groups of animals

Group	Mean frequency (Hz)	Mean amplitude (pA)	Rise time (ms)	Decay time constant (*τ*, ms)	*n*
Tween + NaCl	0.82 ± 0.08	20.02 ± 0.63	1.75 ± 0.11	7.46 ± 0.17	14
Tween + SB	0.83 ± 0.10	19.46 ± 0.69	1.74 ± 0.08	6.58 ± 0.19 *	15
Cort + NaCl	0.27 ± 0.02***	21.00 ± 0.62	2.00 ± 0.07	7.34 ± 0.21	17
Cort + SB	0.83 ± 0.06	19.71 ± 0.72	1.91 ± 0.07	7.27 ± 0.22	18

*n* number of cells

*p < 0.05; ***p < 0.001; Sidak’s multiple comparison test

### Changes in 5-HT_7_ receptor function induced by corticosterone treatment are reversed by SB 269970

**Fig. 4:**
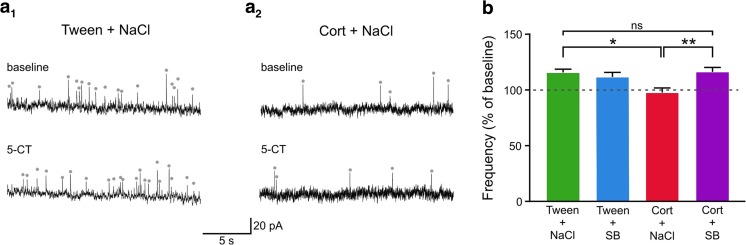
SB 269970 reverses the effect of repeated corticosterone administration on 5-HT_7_ receptor reactivity in the DRN. **a**_**1**_ Sample recordings from a representative neuron in slice prepared from the rat receiving Tween + NaCl before (*upper trace*) and after addition of 250 nM 5-CT to the ACSF (*lower trace*). **a**_**2**_ Sample recordings from a representative neuron in slice prepared from the animal receiving Cort + NaCl before (*upper trace*) and after addition of 250 nM 5-CT to the ACSF (*lower trace)*. Gray dots mark spontaneous synaptic events accepted for analysis. **b** The effect of 5-HT_7_ receptor activation on the sIPSC frequency (mean ± SEM) shown as a percentage of basal sIPSC frequency (before 5-CT addition); *p < 0.05; **p < 0.01; *ns* non-significant effect

A two-way ANOVA revealed a significant interaction between corticosterone and SB 269970 treatments on the 5-CT-induced percentage change in sIPSC frequency (*F*_(1,60)_ = 8.767, *p* = 0.0044). This was not accompanied by significant main effects of either corticosterone treatment (*F*_(1,60)_ = 2.966, *p* = 0.0902) or SB 269970 treatment (*F*_(1,60)_ = 3.329, *p* = 0.0731). The 5-CT-induced increase in sIPSC frequency was similar in the Tween + NaCl and Tween + SB groups (114.14 ± 3.14%, *n* = 14, vs. 116.65 ± 6.66%, *n* = 15 respectively, adjusted *p* = 0.9709, post hoc Sidak’s multiple comparisons test). Corticosterone abolished the 5-HT_7_ effect as there was no 5-CT-mediated increase in sIPSC frequency in the Cort + NaCl group when compared to the Tween + NaCl group (97.49 ± 4.03%, *n* = 17, vs. 114.14 ± 3.14%, *n* = 14 respectively, adjusted *p* = 0.0110, post hoc Sidak’s multiple comparisons test). Co-administration of corticosterone and SB 269970 fully rescued the effect of 5-CT on sIPSC frequency, back to the level seen in the Tween + NaCl group (116.10 ± 3.91%, *n* = 18, vs. 114.14 ± 3.14%, *n* = 14, respectively, adjusted *p* > 0.9999, post hoc Sidak’s multiple comparisons test) (Fig. 4).

There were no significant effects of corticosterone treatment, SB 269970 treatment, or their interaction on 5-CT-induced percentage changes in sIPSC amplitude (main effect of corticosterone treatment: *F*(1, 60) = 0.3492, *p* = 0.8524; main effect of SB 269970 treatment: *F*(1, 60) = 0.5211, *p* = 0.4732; interaction: *F*(1, 60) = 0.1256, *p* = 0.7243), sIPSC rise time (main effect of corticosterone treatment: *F*(1, 60) = 0.1341, *p* = 0.7155; main effect of SB 269970 treatment: *F*(1, 60) = 0.5632, *p* = 0.4559; interaction: *F*(1, 60) = 3.2700, *p* = 0.0756), and sIPSC decay time constant (main effect of corticosterone treatment: *F*(1, 60) = 0.3060, *p* = 0.5822, main effect of SB 269970 treatment: *F*(1, 60) = 0.5458, *p* = 0.4629; interaction: *F*(1, 60) = 1.9230, *p* = 0.1706).

## Discussion

The results of the present study demonstrate that corticosterone treatment lasting 14 days induced a decrease in the frequency of sIPSCs recorded from DRN projection neurons without significant changes in the mean amplitude and kinetic properties, apart from a small decrease in the decay time constant in the case of sIPSCs in cells from the Tween + SB group of animals. These observations are suggestive of a presynaptic location of corticosterone-induced effects on GABAergic transmission within the DRN. In line with this finding, a moderate, chronic increase in circulating corticosterone level has been shown to exert no significant effect on the response of rat DRN 5-HTergic cells to either GABA_A_ or GABA_B_ receptor activation (Judge et al. 2004). In the ventromedial DRN of stressed mice subjected to 5-day social defeat, a decrease in the frequency and a smaller decrease in the amplitude of sIPSCs have been observed (Crawford et al. 2013). It is thus likely that repeated administration of corticosterone brought about a decrease in neurotransmitter release from GABAergic terminals due to a decrease in spiking activity of DRN GABAergic interneurons. It should also be noted that since the DRN receives GABAergic inputs from extrinsic sources including the hypothalamus, substantia nigra, ventral tegmental area, and rostromedial tegmental nucleus (reviewed in: Soiza-Reilly and Commons 2014), it is conceivable that systemic administration of corticosterone reduced the activity of these inhibitory afferent projections as well.

Obtained results demonstrate that repeated corticosterone administration suppressed the reactivity of 5-HT_7_ receptors in the DRN. These receptors appear to be located on DRN GABA interneurons, but not projection neurons, and their activation has earlier been shown to increase the frequency of sIPSCs recorded from DRN 5-HT projection neurons (Mnie-Filali et al. 2011; Kusek et al. 2015), consistent with the fact that 5-HT_7_ receptors’ agonists raise the excitability of the neuron that expresses these receptors (Bacon and Beck 2000; Bickmeyer et al. 2002; Tokarski et al. 2003). Thus, another consequence of prolonged corticosterone exposure is a lack of 5-HT_7_ receptor-mediated indirect inhibitory effect on DRN projection cells. It is tempting to speculate that observed suppression of the reactivity of DRN 5-HT_7_ receptors resulted from enhanced 5-HT release within the DRN. It has been reported that treatment with corticosterone does not result in changes in the basal firing rate of DRN 5-HT neurons or the basal extracellular level of 5-HT in the DRN (Leitch et al. 2003; Rainer et al. 2012). However, GR-mediated reduction of the autoinhibitory function of 5-HT_1A_ somatodendritic receptors in DRN 5-HT neurons has been found to occur after short- and long-term (4–7 weeks) treatment with corticosterone (Fairchild et al. 2003; Laaris et al. 1995; Rainer et al. 2012). Thus, the enhanced 5-HT release is likely to occur in the DRN of corticosterone-treated animals when excitatory inputs to the DRN (reviewed in: Lee et al. 2003) are active. Larger surges of 5-HT within the DRN would result in a stronger, than normal, activation of 5-HT receptors expressed by local neurons, which might lead to their desensitization and/or downregulation. Experiments in vitro have demonstrated that agonist treatment results in a downregulation of 5-HT_7_ receptors in hippocampal neuronal cultures (Vasefi et al. 2013) and desensitization of these receptors in astrocytes of the rat frontal cortex (Shimizu et al. 1998). It should also be noted that 5-HT_7_ receptor functional desensitization is likely to contribute to a decrease in spiking activity of DRN GABAergic interneurons and observed reduction in sIPSCs frequency in DRN projection neurons of corticosterone-treated rats.

It is also conceivable that GR activation might influence the expression level of 5-HT_7_ receptors, as is the case with 5-HT_1A_ receptor in the DRN (Laaris et al. 1995); however, to the best of our knowledge, no data are available on the effects of elevated blood plasma corticosterone on the expression of 5-HT_7_ receptors in the DRN. While adrenalectomy results in an increase of 5-HT_7_ receptor mRNA expression in the hippocampus (Le Corre et al. 1997), we have previously found that repeated administration of corticosterone for 21 days changed neither the affinity (*K*_d_) of hippocampal 5-HT_7_ receptors to [^3^H]-SB 269970 nor their maximum density (*B*_max_, Tokarski et al. 2009).

The present data demonstrate that administration of SB 269970, repeated for 7 days, normalized the frequency of sIPSCs in DRN projection cells of corticosterone-treated rats and restored the reactivity of 5-HT_7_ receptors in the DRN. It has been shown that SB 269970 rapidly enters the brain after intraperitoneal administration and is eliminated within less than 2 h (Hagan et al. 2000). Thus, in our experiments, the blockade of 5-HT_7_ receptors largely coincided with a rapid rise in corticosterone level in the brain which normalizes within 2 h after its subcutaneous administration (Droste et al. 2008). If observed suppression of the reactivity of 5-HT_7_ receptors in DRN GABA interneurons indeed resulted from corticosterone-induced enhancement of 5-HT release within DRN and excessive activation of 5-HT_7_ receptors, their temporal blockade might prevent the occurrence of this effect and lead to the restoration of their reactivity. Interestingly, chronic restraint stress-related hyperactivity of the hypothalamic-pituitary-adrenal axis of rats has recently been linked to an increased expression of 5-HT_7_ receptors in the adrenal cortex (reviewed in: Terrón 2014). It has been reported that SB-656104 (another 5-HT_7_ receptor antagonist) significantly inhibited the sensitized corticosterone response to acute restraint in chronically stressed animals (García-Iglesias et al. 2013). It is conceivable that similar mechanisms might be operable in our experimental model involving repeated corticosterone administration.

Administration of SB 269970 alone, repeated for 7 days, affected neither sIPSCs frequency nor the reactivity of 5-HT_7_ receptors in the DRN. It has been reported that 1-week long treatment of rats with the 5-HT_7_ receptor antagonist SB 269970, in a dose comparable to the one used in the present study, did not alter the firing of DRN 5-HT cells but resulted in desensitization of somatodendritic 5-HT_1A_ autoreceptors, thus providing a prospective fast-acting antidepressant strategy (Mnie-Filali et al. 2011). It should be noted that SB 269970 may differently influence the expression of 5-HT_7_ receptors, as in vitro experiments demonstrated downregulation, upregulation, or lack of effect of SB 269970 on these receptors, depending on a particular splice variant (Andressen et al. 2015). We have demonstrated previously that treatment with SB 269970 induced functional desensitization of 5-HT_7_ receptors in the rat hippocampus (Tokarski et al. 2012b). An earlier study has shown that corticosterone administration lasting 7–21 days enhanced the excitatory effect of 5-HT_7_ receptor activation on the activity of hippocampal pyramidal neurons (Tokarski et al. 2009). Thus, the effects of prolonged corticosterone exposure on hippocampal (Tokarski et al. 2009, 2012b) and DRN (this study) 5-HT_7_ receptors are opposite. However, the results of the present study complement previous work which demonstrated that treatment with SB 269970 counteracts repeated restraint stress-induced detrimental modifications of the glutamatergic transmission and synaptic plasticity in the rat frontal cortex (Tokarski et al. 2011).

Repeated corticosterone administration, as a model of uncontrollable stress, has been suggested to represent a rodent model of depression and anxiety (Zhao et al. 2008; Rainer et al. 2012). The amount of corticosterone administered and the duration of treatment, which produce a depressive-like phenotype, differ between laboratories (reviewed in: Sterner and Kalynchuk 2010). However, repeated corticosterone administration generally produces behavioral and neurobiological alterations that resemble the symptoms and neurobiological changes associated with human depression, including increases in passive behavior and decreases in active behaviors in the forced swim test, inhibited sexual behavior, decreased sucrose intake, decreased responding for food, and decreased grooming (reviewed in: Sterner and Kalynchuk 2010). Importantly, a decrease in 5-HT level by a half in rat frontal cortex has been reported after corticosterone administration in drinking water lasting for 8 weeks (Luine et al. 1993). It should be noted that no change in the basal firing rate of DRN projection cells or extracellular level of 5-HT in the DRN has been found to occur in mice after 7 weeks of corticosterone treatment (Rainer et al. 2012), but this discrepancy might be attributed to species differences. A decreased activity of the serotonergic system has also been found to occur after chronic unpredictable stress, which induces depressive-like phenotype as well (Bambico et al. 2009, reviewed in: Mahar et al. 2014). On the other hand, after administration of corticosterone for 14 days, no change in basal extracellular 5-HT level has been observed in rat hippocampus (Leitch et al. 2003). After 12 days of corticosterone treatment, no change in 5-HT level but a significantly increased amount of 5-HT metabolite 5-hydroxyindoleacetic acid (5-HIAA) in rat frontal cortex has been reported, indicative of an increased metabolism of 5-HT in the DRN target structure (Inoue and Koyama 1996). Thus, corticosterone-induced decrease in the activity of the rat serotonergic system appears to develop only after prolonged exposure to corticosterone, while the early effects may include an increased excitability of DRN projection neurons primarily due to a reduction in the autoinhibitory function of 5-HT_1A_ somatodendritic receptors (Fairchild et al. 2003; Laaris et al. 1995; Rainer et al. 2012) and a reduced GABAergic input from local DRN interneurons (this study). These effects may be accompanied by an elevation of the expression of gene encoding tryptophan hydroxylase 2 (*tph2*), the rate-limiting enzyme for brain 5-HT synthesis in the DRN, reported to occur after 3 weeks of treatment of rats with corticosterone (Donner et al. 2012).

## Conclusion

In conclusion, the results of the present study indicate that repeated administration of exogenous corticosterone induces a reduction of 5-HT_7_ receptor reactivity and weakening of inhibitory transmission within the DRN neuronal network. Administration of a 5-HT_7_ receptor antagonist restores 5-HT_7_ receptor reactivity and reverses the effects of elevated corticosterone levels on inhibitory transmission within the DRN. These findings may be of importance for the treatment of stress-related disorders.
